# Factors Associated with Adherence to Recommended Colorectal Surveillance Intervals in Lynch Syndrome

**DOI:** 10.3390/cancers18122010

**Published:** 2026-06-22

**Authors:** Danielle Mirda, Jinxuan Hao, Michaela Dungan, Julia Youngman, Yue Ren, Hongzhe Li, Jessica M. Long, Bryson W. Katona

**Affiliations:** 1 Department of Medicine, Hospital of the University of Pennsylvania, Philadelphia, PA 19104, USA; daniellemirda94@gmail.com (D.M.); jinxuan.hao@pennmedicine.upenn.edu (J.H.); 2 Division of Gastroenterology and Hepatology, Perelman School of Medicine, University of Pennsylvania, Philadelphia, PA 19104, USA; michaela.dungan@pennmedicine.upenn.edu (M.D.); julia.youngman@pennmedicine.upenn.edu (J.Y.); 3 Department of Biostatistics, Epidemiology, and Informatics, Perelman School of Medicine, University of Pennsylvania, Philadelphia, PA 19104, USA; yueren@pennmedicine.upenn.edu (Y.R.); hongzhe@pennmedicine.upenn.edu (H.L.); 4 Division of Hematology-Oncology, Department of Medicine, Penn Medicine, Philadelphia, PA 19104, USA; jessica.long@pennmedicine.upenn.edu; 5 King Center for Lynch Syndrome, Abramson Cancer Center, Penn Medicine, Philadelphia, PA 19104, USA

**Keywords:** Lynch syndrome, colorectal cancer, surveillance adherence

## Abstract

**Background**: Lynch syndrome (LS) is the most common hereditary cause of colorectal cancer (CRC), and colorectal surveillance has been shown to reduce death from CRC in this high-risk population. This study aimed to examine adherence to recommended endoscopic surveillance intervals in a LS cohort and identify factors associated with adherence. **Methods**: A retrospective review was performed of 1403 lower endoscopic procedure (LEP) reports from 540 individuals with LS completed between May 2001 and September 2023. Adherence was calculated by comparing the endoscopists’ recommended interval to the actual date of the subsequent procedure. Reports from individuals with ≥2 LEPs and those with follow-up intervals within the study period were included (n = 1170). A mixed-effect logistic regression analysis identified factors associated with adherence. **Results**: Among 1170 LEPs from 295 LS carriers, 67.4% were performed within the recommended interval. However, 68.8% of individuals had at least one procedure that was delayed. Increased adherence was significantly associated with prior detection of CRC (OR 9.30, 95% CI 1.16–74.32, *p* = 0.035) on an LEP. Marital status was significantly associated with adherence, with higher rates among married (OR 1.73, 95% CI 1.05–2.85, *p* = 0.030) and divorced/widowed individuals (OR 2.32, 95% CI 1.14–4.72, *p* = 0.020). Current smokers had a lower rate of adherence (OR 0.33, 95% CI 0.13–0.82, *p* = 0.018). No significant associations were observed with age, biological sex, race, or insurance status. **Conclusions**: Nearly one third of LEPs in our LS cohort were delayed. Efforts to address barriers and enhance adherence are essential to optimize outcomes for this high-risk population.

## Simple Summary

Lynch syndrome is the most common inherited cause of colorectal cancer, and regular colonoscopy surveillance is critical to reduce cancer risk and mortality. In this study, we examined how often individuals with Lynch syndrome adhere to recommended surveillance timing and identified factors associated with adherence. We found that nearly one third of procedures were delayed, with most patients experienced at least one delay. Individuals with a prior diagnosis of colorectal cancer or advanced precancerous polyps were more likely to adhere to surveillance, while current smokers were less likely to do so. Marital status was also associated with adherence, suggesting a role for social support. These findings highlight the need for targeted strategies to improve adherence, particularly among higher-risk and underserved groups, to optimize cancer prevention outcomes.

## 1. Introduction

Colorectal cancer (CRC) is the second leading cause of cancer-related death in the United States [1]. Lynch syndrome (LS), the most common hereditary cause of CRC, accounts for 2–5% of CRC cases and carries a cumulative lifetime CRC risk of up to 70% [2,3]. LS results from pathogenic germline variants (PGVs) in DNA mismatch repair (MMR) genes, leading to the early development of CRCs that arise from colorectal adenomas and/or MMR-deficient crypts, and are characterized by microsatellite instability and a high tumor mutational burden [4,5,6]. However, CRC risk varies within LS, with *MSH6* and *PMS2* PGV carriers having a lower risk compared to those with PGVs in *MLH1*, *MSH2*, or *EPCAM* [4]. Given the elevated risks, CRC surveillance is a critical component of a LS cancer risk management plan and has been shown to decrease the risk of CRC and decrease mortality from CRC in this particularly high-risk population [7,8]. Conversely, not adhering to surveillance may carry meaningful consequences: a recent retrospective cohort study conducted in Italy demonstrated that individuals with LS who did not complete surveillance had significantly higher rates of CRC diagnoses compared to those who completed surveillance (19.6% vs. 11.5%, *p* = 0.036) [9], highlighting the importance of regular endoscopic follow-up.

With a lack of international consensus on endoscopic surveillance guidelines, recent discussion has focused on determining the most appropriate endoscopic surveillance intervals for individuals with LS. Some studies report no significant differences in CRC incidence and mortality between shorter and longer surveillance intervals [10,11,12], whereas other studies demonstrate substantial CRC risk reductions with shorter surveillance intervals, especially for high-risk PGVs [7,13]. In the United States, current National Comprehensive Cancer Network (NCCN) guidelines reflect the variability in CRC risk, recommending endoscopic surveillance in LS every 1–2 years starting at age 20–25 for high-risk PGV carriers (*MLH1*, *MSH2*, and *EPCAM*) and every 1–3 years starting at age 30–35 for *MSH6* and *PMS2* carriers [14]. With guidelines establishing these surveillance recommendations, ensuring patient adherence to surveillance recommendations remains a critical component of managing cancer risk for individuals with LS.

There have been several studies evaluating surveillance compliance in LS, reporting per-patient adherence rates ranging from 72% to 88% and per-procedure adherence rates of 68% to 80% [9,12,15,16,17,18,19]. Factors associated with adherence noted in these studies were sex, age, and personal history of CRC, although data remain mixed [7,15,16]. The majority of the published studies are from Europe, with only two small U.S.-based studies that have examined adherence, reporting rates between 73% and 80% [12,15]. Notably, European healthcare systems, such as those in Finland and the Netherlands, function with centralized cancer registries that facilitate surveillance, whereas in the U.S., this responsibility often falls on individual healthcare providers [17,20]. Thus, beyond patient-specific characteristics, these systemic differences limit the generalizability of European data to U.S. populations, underscoring a critical gap in the literature. To address this, we conducted a cohort study of a LS cohort to evaluate overall adherence to colorectal surveillance and identify factors influencing adherence among individuals with LS in the United States.

## 2. Materials and Methods

### 2.1. Study Population

This study was approved by the Institutional Review Board of the University of Pennsylvania. In this retrospective study, we identified individuals with LS who were evaluated at Penn Medicine between May 2001 and September 2023. All included individuals had a confirmed PGV in *MLH1*, *MSH2*, *MSH6*, *PMS2*, or *EPCAM*, or were an obligate carrier of a PGV in one of these genes.

### 2.2. Study Design

Demographic data, personal medical history, family history, and colonoscopy/sigmoidoscopy reports and associated pathology were collected and entered into a secure REDCap database. Only lower endoscopic procedures (LEPs) performed after LS diagnosis, and those conducted between May 2001 and September 2023, were reviewed. Notably, LEPs refer to both colonoscopies and sigmoidoscopies, and all sigmoidoscopies performed were conducted in individuals with a prior history of colonic resection. A retrospective review of 1403 colonoscopy and sigmoidoscopy reports from 540 individuals was performed, including procedures conducted at both Penn Medicine and other community endoscopy centers. Data from individuals who had undergone at least two surveillance colonoscopies or sigmoidoscopies (n = 295) were included. Only LEPs with a recommended surveillance interval falling within the study period were included (n = 1170).

### 2.3. Study Analysis

The primary outcome of this study was adherence to lower endoscopic surveillance recommendations. This was calculated as a rate by comparing the interval recommended in the endoscopy report to the actual date the subsequent procedure was performed. A 3-month grace period beyond the recommended interval was permitted to account for scheduling delays and/or insurance factors. The secondary outcome was to identify factors associated with adherence.

Baseline demographic and clinical characteristics were first compared between adherent and non-adherent individuals (defined as those with all versus at least one delayed LEP, respectively) using chi-square or Fisher’s exact tests for categorical variables and a Wilcoxon rank-sum test for continuous variables, given non-normal distributions. To identify factors independently associated with adherence at the procedure level, a mixed-effects logistic regression analysis was performed with adherence of each LEP as the outcome and individual patient as the random effect, accounting for the correlation of multiple procedures nested within the same patient. Results for this analysis are reported as odds ratios with 95% confidence intervals. Statistical analyses were conducted using R software (version 4.2.2). A *p*-value < 0.05 was considered statistically significant.

## 3. Results

### 3.1. Cohort Demographics and Characteristics

**Figure 1:**
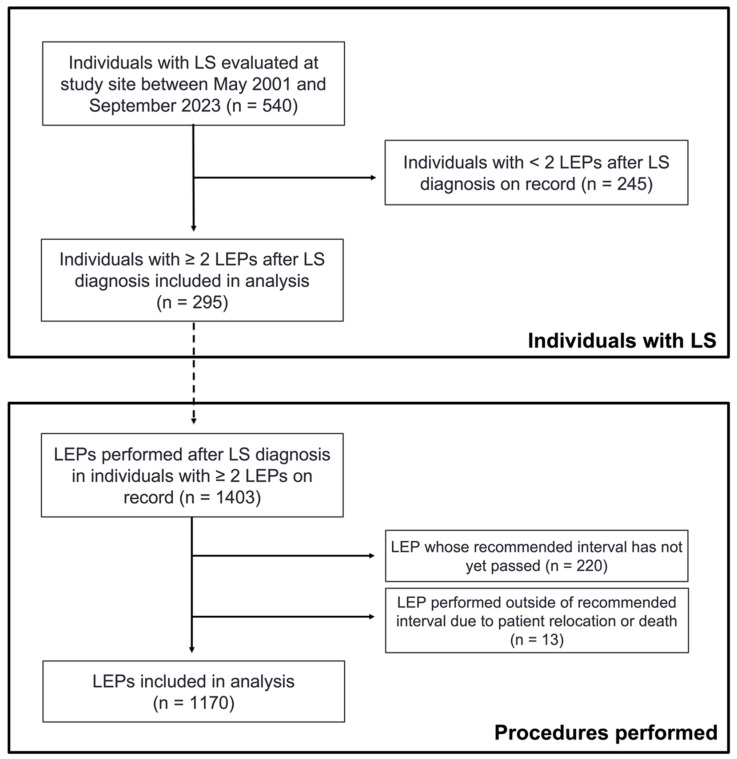
Flowchart of individuals with LS evaluated at the study site and LEPs included in the analyses.

A total of 540 individuals with LS were identified during the study period. Of these, 245 individuals (43.9%) were excluded due to having fewer than two LEPs on record that occurred after their LS diagnosis (Figure 1). This excluded group primarily included individuals who had only one post-diagnosis LEP, many of whom were more recently diagnosed with LS and had not yet reached their next recommended surveillance interval. Additionally, some had procedures performed at outside institutions with inaccessible records or were seen only once for consultation, and, therefore, longitudinal data were not available. Consequently, 295 individuals met the inclusion criteria and were included in the study analysis.

A total of 1403 LEPs were performed after a LS diagnosis in individuals with at least two LEPs on record during the study period. LEPs were excluded if the recommended interval had not yet passed (n = 220) or if the reason for delayed surveillance was patient relocation or death (n = 13). Ultimately, a total of 295 individuals and 1170 colonoscopies and sigmoidoscopies were included in the analyses (Figure 1). Of note, of the 1170 LEPs, 1014 (86.7%) were colonoscopies, and 156 (13.3%) were sigmoidoscopies. A total of 85.9% of procedures were completed at Penn Medicine, and 14.1% were completed at outside endoscopy centers.

The cohort was primarily White (88.5%), female (62.7%), married (70.8%), privately insured (75.3%), and had never smoked (69.5%) (Table 1). The median age at first LEP after LS diagnosis was 49 years (interquartile range [IQR], 38–61). The most common genotype was *MSH2/EPCAM* (35.6%), followed by *MLH1* (23.1%), *PMS2* (19.7%), and *MSH6* (21.7%). A prior history of CRC was present in 23.1% of the cohort, 29.5% had undergone a prior colorectal resection, and 33.9% had a history of a non-colorectal malignancy. Aspirin use for ≥2 years was reported in 44.4% of individuals, and the median endoscopic surveillance interval was 1.1 years (IQR, 1.0–1.6). When stratified by adherence status, adherent and non-adherent individuals were largely similar across all baseline characteristics, including genotype distribution (*p* = 0.53), with no statistically significant differences identified except for the median time since last LEP, which was shorter among adherent compared to non-adherent individuals (1.0 vs. 1.2 years, *p* < 0.01) (Table 1).

### 3.2. Adherence to Surveillance

**Figure 2:**
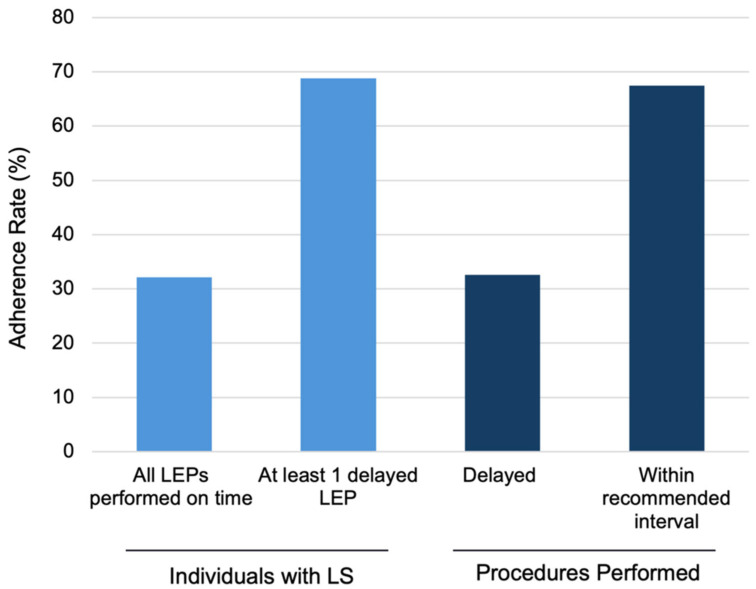
Adherence to surveillance intervals stratified by individuals with LS and procedures performed.

Of the 1170 LEPs reviewed across 295 individuals with LS, 67.4% were performed within the recommended surveillance interval (Figure 2). Of the 381 delayed procedures, 53 had no follow-up procedure completed within the study period and no future procedure scheduled, indicating these individuals were lost to follow-up. For the remaining 328 delayed procedures with available follow-up data, the median delay was 0.55 years (6.6 months), and the mean delay was 0.80 years (9.6 months). Among these, 90 had a subsequent surveillance procedure scheduled but not yet completed at the end of the study period. Of the remaining 237 delayed procedures that were completed during the study period, five CRCs (2.1% of delayed procedures) and 10 advanced adenomas (4.2% of delayed procedures) were identified, with no advanced serrated lesions identified. Only 31.2% of individuals had adhered to the recommended surveillance interval across all colonoscopies or sigmoidoscopies; therefore, 68.8% of individuals had at least one surveillance LEP that was delayed.

### 3.3. Factors Associated with Adherence

**Figure 3:**
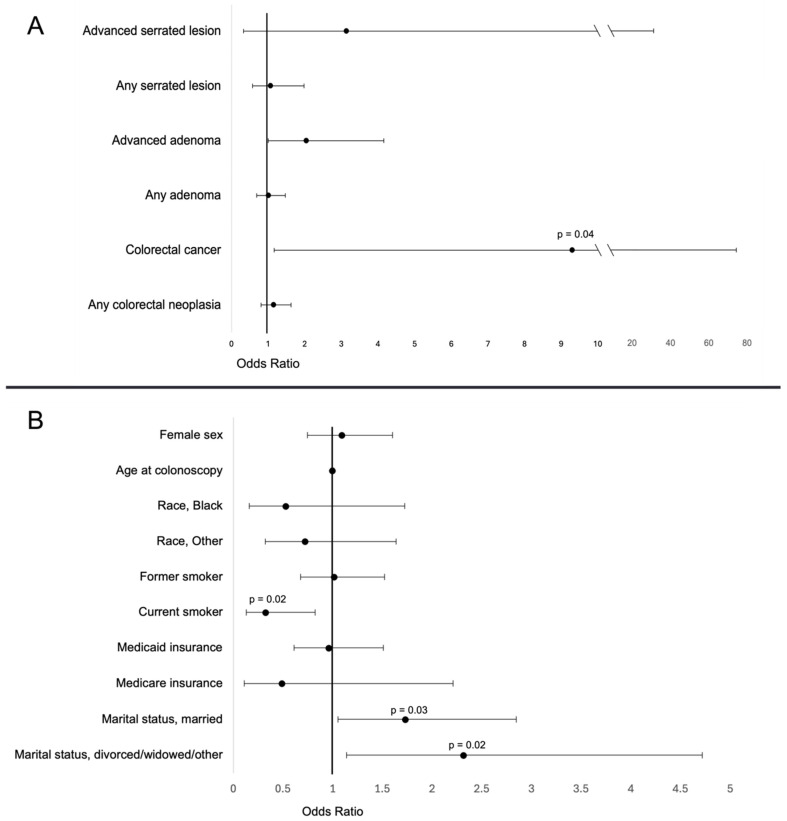
Association of adherence to recommended surveillance interval with (**A**) prior neoplasia detection and (**B**) demographic risk factors.

We next assessed for clinical and demographic factors that may be associated with surveillance adherence (Table 2). Having a prior LEP that detected CRC (OR 9.30, 95% CI 1.16–74.32, *p* = 0.035) was significantly associated with increased adherence to surveillance recommendations (Figure 3A). Marital status was also significantly associated with adherence. Compared to those who were single, individuals who were married (OR 1.73, 95% CI 1.05–2.85, *p* = 0.030), as well as those who were divorced or widowed (OR 2.32, 95% CI 1.14–4.72, *p* = 0.020), had increased adherence to LEP surveillance (Figure 3B). Current smokers had lower rates of LEP adherence (OR 0.33, 95% CI 0.13–0.82, *p* = 0.018) compared to never or former smokers. No statistically significant associations were found with age, biological sex, race, or insurance status.

Among records where a reason for delayed surveillance was documented, commonly cited factors included incomplete bowel preparation or bowel preparation intolerance, fear of illness due to COVID-19, missed or canceled appointments, and non-responsiveness to scheduling attempts.

## 4. Discussion

Regular endoscopic surveillance is essential for preventing CRC morbidity and mortality in individuals with LS, yet there are limited U.S.-based studies evaluating adherence to recommended surveillance intervals. Studying adherence to endoscopic surveillance in LS is critical to understand factors contributing to these high-risk individuals not obtaining the life-saving colorectal surveillance that is recommended. Therefore, in this retrospective cohort study of 295 LS carriers and 1170 lower endoscopic procedures, we assessed adherence rates and identified factors influencing adherence. Although large multicenter studies have investigated surveillance adherence in LS cohorts in Europe [9,12,17,18,19], this study is among the few conducted in the United States and represents the largest to date [15,16], addressing a critical gap in the literature.

In our LS cohort, 67.4% of colonoscopies and sigmoidoscopies were performed within the recommended surveillance intervals, but only 31.2% of individuals had optimal adherence across all their surveillance LEPs. These adherence rates are lower than those reported in two prior U.S.-based studies. The first, conducted by Mittendorf et al. within the Kaiser Permanente Northwest system, found that 80% of surveillance intervals were compliant [16]. This higher rate may be attributed to the integrated nature of the Kaiser health system, which likely reduces barriers and delays. The second study found that 73% of patients were compliant with endoscopic surveillance [15]; however, this was based on self-reported survey response data, which introduces significant bias and may overestimate true adherence. A recently published retrospective study from Kaiser Permanente Northern California that examined rates of NCCN guideline-concordant screening in LS found that while 59% of their cohort underwent at least one colonoscopy, only 40% did so at the appropriate interval [21]. The authors attribute suboptimal adherence in part to provider-level factors, including familiarity with LS recommendations among primary care providers. In our practice setting, where individuals with LS are followed by a multidisciplinary team with expertise in LS, provider unfamiliarity is less likely to account for non-adherence, suggesting that patient and system-level barriers are likely the dominant drivers in our cohort. Collectively, these findings underscore the suboptimal adherence across diverse healthcare systems and highlight the value of future multicenter collaboration.

Several factors were found to be significantly associated with increased adherence to surveillance in our study. Notably, detection of a CRC on a prior surveillance LEP was associated with higher adherence rates. This likely reflects both the motivating effect of a prior CRC diagnosis in reinforcing the life-saving potential of surveillance and the tendency for individuals who have experienced a serious diagnosis to be more inclined to follow surveillance guidelines. However, prior studies have reported mixed results regarding the association between a personal history of CRC prior to LS diagnosis and adherence rates [15,16]. Marital status was also associated with increased adherence; individuals who were married or previously married (i.e., divorced/widowed) had statistically significantly higher adherence rates than those who were single. This suggests that social support may play a role in promoting adherence to recommendations. A variable not collected during the study was whether the individual had children, which may be more common among those who are married, divorced, or widowed. Future studies could explore whether having children is also associated with increased surveillance adherence.

Current smoking was associated with lower rates of adherence, a finding consistent with studies conducted in the general population, which show that smokers tend to have lower engagement in preventive healthcare and lower rates of cancer screenings, including colonoscopy [22,23]. This has been thought to be due to several factors, including more pessimistic and avoidant beliefs about cancer, various socioeconomic determinants, and healthcare avoidance due to fear or stigma [23,24]. These findings emphasize the need for targeted interventions to improve surveillance adherence among smokers, particularly in individuals with LS.

While other researchers have reported that younger patients and females were more likely to be adherent [7], we did not find significant associations between adherence and age, biological sex, race, or insurance status. This discrepancy may be attributed to the demographic nature of our cohort, which was predominantly White and privately insured. This likely limited our statistical power to detect meaningful differences across racial and socioeconomic groups and may obscure disparities that are well-documented in the broader colorectal cancer screening literature, including barriers related to access to genetic counseling, language discordance, and systemic healthcare inequities [25,26].

Primary reasons for non-adherence in our study were infrequently documented, but when available, patient-centric factors were most commonly cited. These included challenges such as intolerance to bowel preparation, fear of illness due to COVID-19, non-responsiveness to scheduling attempts by staff, and scheduled appointments that were not completed. Similarly, a prior survey study assessing attitudes toward colonoscopy and adherence in LS carriers found that bowel preparation was perceived as the most burdensome aspect of surveillance, with delays primarily attributed to patients not scheduling their procedure far enough in advance or postponing the procedure [19]. Other studies have highlighted poor communication from the healthcare system as a barrier, noting that reminder letters significantly improved compliance [17,27]. These findings underscore the need to address patient concerns and logistical challenges to improve adherence rates. Specifically, efforts to mitigate preparation intolerance and streamline scheduling processes could further enhance adherence.

Several system-level interventions have shown promise in improving surveillance adherence in LS and CRC more broadly. Recent work demonstrated that an LS-specific clinical decision support tool can be built into the electronic medical record to help promote surveillance adherence [28]. Patient navigation has also emerged as a promising strategy for lessening multiple barriers to CRC screening, particularly in Black and Latino populations, by addressing education gaps as well as logistical and communication challenges [29,30], and may be beneficial in LS patients as well. While our study focused primarily on patient-level characteristics, future prospective studies should incorporate a systematic evaluation of healthcare system factors, such as EMR reminders and institutional tracking systems, in addition to patient surveys and patient navigation models, to more completely characterize barriers to adherence and inform targeted strategies to improve surveillance in LS.

Limitations of this study include its retrospective design, which may have led to incomplete data collection, particularly regarding reasons for non-adherence. Importantly, as a single-center study conducted at a tertiary academic medical center, our findings may not be generalizable to the broader LS population. Given that tertiary care centers typically serve patients with greater access to specialized genetic counseling and surveillance programs, our adherence estimates may have been inflated compared to community or safety-net settings. Future multicenter prospective studies incorporating diverse geographic regions and demographic populations, including underrepresented racial and ethnic minorities, rural communities, and patients of varying socioeconomic backgrounds, are needed to more accurately characterize surveillance adherence and identify barriers at a population level. Procedures conducted at outside community endoscopy centers were included, but incomplete records due to incompatible electronic medical record systems may have led to an underestimation of adherence. Our analysis focused on individuals with at least two post-LS diagnosis LEPs, which may have enriched the study population with more adherent individuals. While many excluded individuals were recently diagnosed with LS and not yet due for a second LEP, others may reflect early attrition or loss to follow-up.

Additionally, the 22-year data collection period spans several important changes that may have influenced adherence rates. First, surveillance recommendations for LS evolved considerably over this time period. Early in the study timeframe, guidelines recommended uniform colonoscopy every 1–2 years starting at age 20–25 for all LS carriers regardless of genotype, whereas more recent gene-specific guidelines recommend later initiation and longer intervals between colonoscopies for lower-penetrance *MSH6* and *PMS2* carriers [14], which may have altered surveillance schedules as well as patient behavior over time during the study period. Advances in endoscopic technology have also improved adenoma detection rates, which may prompt recommendations for more frequent surveillance. Lastly, changes in healthcare delivery, particularly the widespread adoption of electronic health records (EHRs), may have impacted adherence as EHR-based reminder systems and clinical decision support tools have been shown to facilitate surveillance in LS patients [28]. Future prospective studies that incorporate a broader, more diverse cohort and explore specific interventions to improve adherence would provide valuable insights into how to best support individuals with LS in adhering to recommended surveillance procedures.

## 5. Conclusions

While a majority of colonoscopies and sigmoidoscopies in individuals with LS were completed within the recommended surveillance interval, nearly one third were delayed. Key factors influencing adherence included a history of high-risk lesions detected on a prior LEP, marital status, and smoking behaviors. These findings underscore the need for targeted interventions to improve surveillance adherence, particularly among those who smoke, those without a robust social support structure, and those who have not had prior surveillance-detected colonic neoplasia. Future prospective studies with diverse cohorts are needed to explore effective strategies for enhancing adherence to surveillance recommendations and ultimately improving health outcomes for individuals with Lynch syndrome.

## Figures and Tables

**Table 1:** Cohort of individuals with LS where colorectal surveillance adherence was evaluated.

Variable	Overall	Adherent	Non-Adherent	*p*
N	295	92	203	
Age at first LEP after LS diagnosis (median [IQR])	49.2 [37.8, 61.1]	50.0 [40.2, 60.4]	48.4 [36.9, 62.1]	0.46
Biological sex (%)				0.12
Male	110 (37.3)	27 (29.3)	83 (40.9)	
Female	186 (63.0)	65 (70.7)	120 (59.1)	
Race (%)				
White	261 (88.5)	84 (91.3)	177 (87.2)	0.27
Black	8 (2.7)	2 (2.2)	6 (3.0)	
Other	26 (8.8)	6 (6.5)	20 (9.8)	
Gene (%)				0.53
* MLH1*	68 (23.1)	22 (23.9)	46 (22.7)	
* MSH2/EPCAM*	105 (35.6)	28 (30.4)	77 (37.9)	
* PMS2*	58 (19.7)	18 (19.6)	40 (19.7)	
* MSH6*	64 (21.7)	24 (26.1)	40 (19.7)	
Marital status (%)				0.67
Single	53 (18.0)	15 (16.3)	38 (18.7)	
Married	209 (70.8)	65 (70.7)	145 (71.4)	
Divorced/Widowed/Other	33 (11.2)	12 (13.0)	20 (9.9)	
Insurance type (%)				0.78
Private	222 (75.3)	71 (77.2)	151 (74.4)	
Medicare	65 (22.0)	19 (20.7)	46 (22.7)	
Medicaid	6 (2.0)	2 (2.2)	4 (2.0)	
Other	2 (0.7)	0 (0.0)	2 (1.0)	
Smoking status (%)				0.69
Never	205 (69.5)	67 (72.8)	138 (68.0)	
Former	78 (26.4)	22 (23.9)	56 (27.6)	
Current	12 (4.1)	3 (3.3)	9 (4.4)	
BMI at first LEP after LS diagnosis (median [IQR])	26.6 [24.2, 30.5]	26.3 [23.4, 29.9]	26.8 [24.5, 30.7]	0.18
ASA use ≥2 years (%)	131 (44.4)	39 (42.4)	92 (45.3)	0.73
Years since last LEP (median [IQR])	1.1 [1.0, 1.6]	1.0 [1.0, 1.1]	1.2 [1.0, 1.9]	<0.01
History of prior colon resection (%)	87 (29.5)	26 (28.3)	61 (30.0)	0.86
Personal history of any prior cancer (%)	168 (56.9)	53 (57.6)	115 (56.7)	0.98
Personal history of prior CRC (%)	68 (23.1)	19 (20.7)	49 (24.1)	0.61
Personal history of prior non-CRC (%)	100 (33.9)	34 (37.0)	66 (32.5)	0.54
Family history of any cancer (%)	288 (97.6)	89 (96.7)	199 (98.0)	0.79
Family history of CRC (%)	229 (77.6)	71 (77.2)	158 (77.8)	1.00
Family history of non-CRC (%)	59 (20.0)	18 (19.6)	41 (20.2)	1.00

**Table 2:** Factors associated with adherence to recommended colorectal surveillance intervals.

Variable	Odds Ratio (95% CI)	*p*-Value
***Neoplasia-related factors***		
Personal history of prior cancer before LS diagnosis		
None	1	Ref
Any cancer	1.373 (0.945, 1.995)	0.096
Colorectal cancer	1.092 (0.723, 1.65)	0.677
Other cancer	1.373 (0.953,1.978)	0.183
Family history of cancer		
None	1	Ref
Any cancer	1.343 (0.374, 4.829)	0.651
Colorectal cancer	1.367 (0.869, 2.149)	0.176
Other cancer	1.454 (0.772, 2.738)	0.247
Colorectal neoplasia detected after LS diagnosis		
None	1	Ref
Any colorectal neoplasia	1.145 (0.806, 1.626)	0.45
Colorectal cancer	9.302 (1.164, 74.32)	0.035
Advanced or non-advanced adenoma	1.004 (0.686, 1.468)	0.984
Advanced adenoma	2.038 (0.10, 4.155)	0.05
Advanced or non-advanced serrated lesion	1.064 (0.573, 1.978)	0.844
Advanced serrated lesion	3.136 (0.329, 29.89)	0.321
***Demographic-related factors***		
Biological Sex		
Male	1	Ref
Female	1.092 (0.744, 1.601)	0.654
Age at colonoscopy	0.998 (0.985, 1.011)	0.720
Race		
White	1	Ref
Black	0.528 (0.162, 1.723)	0.290
Other	0.724 (0.32, 1.637)	0.438
Marital Status		
Single	1	Ref
Married	1.731 (1.053, 2.847)	0.030
Divorced/Widowed/Other	2.318 (1.139, 4.719)	0.020
Insurance type		
Private	1	Ref
Medicare	0.961 (0.612, 1.51)	0.865
Medicaid	0.489 (0.108, 2.212)	0.353
Smoking Status		
Never	1	Ref
Former	1.014 (0.675, 1.522)	0.948
Current	0.326 (0.129, 0.822)	0.018

## Data Availability

The datasets used and/or analyzed during the current study are available from the corresponding author on reasonable request.

## Abbreviations

The following abbreviations are used in this manuscript:

CRC	Colorectal cancer
LS	Lynch syndrome
LEP	Lower endoscopic procedure
